# Masculinities and Social Contexts of HIV Risk Practices Among Central Asian Male Migrant Workers

**DOI:** 10.4172/2155-6113.1000486

**Published:** 2015

**Authors:** Nabila El-Bassel, Stacey A Shaw, Gaukhar Mergenova, Leyla Ismayilova, Tara McCrimmon, Assel Terlikbayeva, Louisa Gilbert

**Affiliations:** 1 Global Health Research Center of Central Asia, Columbia University School of Social Work, 1255 Amsterdam Ave, 8th Floor, New York, NY, USA; 2 Global Health Research Center of Central Asia, 102 Luganskogo St, ap.1, Medeu district, Almaty, Kazakhstan; 3 School of Social Service Administration, University of Chicago, 969 E. 60th St., Chicago, IL, USA

**Keywords:** Migration, Central Asia, Sexual risk, HIV, Masculinity

## Abstract

**Background:**

Migrant workers worldwide are at high risk of acquiring and transmitting HIV and sexually transmitted infections. Over the past decade Central Asia has experienced an increase in new HIV infections and migration and mobility within the region. These trends call for mixed methods research to explore the environmental and mobility contexts in which HIV risk behaviors occur among Central Asian migrants, particularly those in Kazakhstan, a common destination country.

**Methods:**

This study took place in Almaty’s Baraholka Market, which employs 30,000 workers including many migrant workers from Central Asian countries. We used a convenience sampling approach to recruit 48 male migrant workers from Uzbekistan, Tajikistan, Kyrgyzstan, and Kazakhstan. Through in-depth interviews, we examined both their engagement in a number of sexual HIV risk behaviors (including having outside partners, sex trading, and condom use) as well as the meaning they attributed to such behaviors. We also address micro-social contexts (employment, types of relationships, infidelity, and access to resources), macro-contexts (gender roles and power dynamics) and structural contexts (mobility and policing) to examine how environmental influences influenced HIV risks.

**Results:**

Findings suggest that men in this study attributed sex with extramarital partners to sexual desires when wives are unable to have sex, mobility and separation, need for variety, and lack of satisfaction in marriage. Factors influencing condom use included trust, sexual pleasure, intention to protect one’s health, and alcohol use. Participants had low levels of knowledge about HIV/AIDS; believed HIV did not affect their community or social networks, and had limited access to health care.

**Conclusions:**

Study findings call for a combination of contextually sustainable HIV/STI prevention strategies that target migrant workers in Central Asia.

## Background

Although worldwide incidence rates of HIV fell between 2001 and 2009, in seven countries including Kazakhstan, Kyrgyzstan, and Tajikistan, HIV incidence rates increased more than 25% [1]. Injection drug use has been the primary driver of new infections in Central Asia, but migrants, along with commercial sex workers, men who have sex with men, and people with a history of incarceration are also at increased risk [2,3].

Despite the large number of migrant workers in Kazakhstan, little is known about their HIV risk environment. Kazakhstan is the 15th top destination country for migrants worldwide and is second only to Russia in receiving migrants from other Central Asian countries [4], hosting approximately 3.5 million migrants [5]. A recent study conducted among migrant market vendors in Kazakhstan demonstrated high rates of unprotected sex and sex with multiple partners among a sample of 250 men [6]. Mobility was identified as a risk factor, as those who traveled more often were more likely to have multiple sexual partners and to have recently visited a sex worker [6]. Throughout the region, studies have found low levels of HIV awareness among migrants [2,7]. Another study found high rates of sexual risk behaviors among male Central Asian migrants working in Moscow, with approximately 90% of men reporting commercial partners and less than 10% reporting regular condom use with their main partners or wives [8]. To date, however, there remains very limited understanding of the attitudes, beliefs and wider environmental and socio-cultural contexts that influence sexual risk behaviors among migrant men in Central Asia.

## Objective

This qualitative study identifies the contexts in which HIV risk occurs among 48 male migrant market workers recruited from the Baraholka marketplace in Almaty, Kazakhstan. We used in-depth interviews to examine participants’ socio-demographic characteristics, employment, living arrangements, micro-social contexts (employment, types of relationships, infidelity, and access to resources), macro-contexts (gender roles and power dynamics) and structural contexts (mobility and policing), and sexual HIV risk-taking, as well as the meanings participants attach to specific risks. Findings from these in-depth interviews elucidate migrants’ HIV risks through their own accounts and interpretations of the sexual behavior and risk environments affecting their behaviors. We also discuss the implications of these findings for HIV prevention services.

This paper is guided by the theory of hegemonic masculinity [9], which has been used to understand variations of masculinities that occur in different social contexts [10] among men including male migrant workers as they move to new destinations and social spaces [11]. The concept of hegemonic masculinities by Connell proposes that not all men benefit equally from institutions of patriarchy and that some types or forms of masculinities are culturally promoted or respected more than others [9]. The idea of hegemonic masculinities reflects the power dimensions of gender relations, marginalization, and subordination of men [12]. The hegemonic masculinities framework can be used to explain how migrant men negotiate, react, and respond to male identities that they encounter within their own groups and others. Migrant men bring beliefs and well-established behaviors about manhood and gender relations with them to new destinations. Beliefs about their lifestyle, HIV, sexuality, and sexual risks are challenged, reproduced, justified, or reinforced in new environments where they face numerous challenges, stigmatization, and marginalization as migrant workers [9]. This paper provides data on masculinities in relation to sexual HIV risk behaviors, as we examine the meanings of men’s accounts through the lens of this theory.

## Materials and Methods

Forty-eight in-depth interviews were conducted as a qualitative component of the Silk Road Health Project, a longitudinal epidemiological study examining contexts of HIV risk among market vendors in Kazakhstan, and were completed in 2010. The Columbia University Institutional Review Board approved all study procedures and provided ethical clearance for this study. Participants were recruited from Baraholka Market, the largest market in Central Asia, which contains 28 independent submarkets, with approximately 17,500 stalls and 30,000 workers. Men were eligible if they were between the ages of 18 and 50, worked in the market, reported having had vaginal or anal sex with a woman in the past 90 days, and were from Uzbekistan, Tajikistan, Kyrgyzstan, Kazakhstan, or were ethnically Uighur. Participants also needed to indicate that they had a permanent residence outside of Kazakhstan or beyond a two-hour commuting distance from Almaty and to report crossing an international border in the past year. Spoken Russian or Kazakh proficiency was also required for eligibility.

### Recruitment procedures

We used a convenience sampling approach. Trained research assistants approached market workers and provided fliers and descriptive information about the study. Potential participants were informed that the general purpose of the study was to discuss their knowledge about migrant market workers’ experiences, and that all information provided would be confidential (not shared with police or market administration). Those interested in participating provided initial consent and were given a five-minute screening interview. If the participant was eligible, he was asked to complete the informed consent process to participate in the interview, including permission to audio-record the interview. Interviews took place in the project office or another private location selected by the participant. Each participant was compensated with approximately US $10.

### Measurement

In-depth interviews examined cultural meanings, values, beliefs, gender norms, and power dynamics that may influence sexual risk behavior, with specific questions about migration experiences, living situations, social dynamics, and risk behaviors. The interview guide included questions regarding the participant’s socio-demographic characteristics; living arrangements; migration and mobility experiences; working conditions and socializing; views on relationships, sex, intimacy, and monogamy; HIV/AIDS/STI awareness; mobility and sex while travelling; access to health care and services; and religion. Bilingual, trained researchers conducted the interviews, which were audio-recorded and transcribed verbatim into Russian and Kazakh. Interviews were then translated into English by a different bilingual researcher, and back-translated into Russian or Kazakh to ensure accuracy.

### Analytic approach

After translation and transcriptions were complete, each interview was summarized and the research team reviewed initial emerging themes. Research assistants in collaboration with study Principal Investigators then engaged in first cycle coding methods including structural and magnitude coding to identify content related to sexual risk and migration. This thematic analysis approach included immersion in the data, generating initial codes, and identifying themes [13]. A list of 14 primary codes (such as ‘partners,’ ‘condoms,’ ‘health,’ and ‘HIV/STIs’) was agreed upon, after which two researchers coded all interviews using NVivo 10 software and identified other emerging categories. Inter-rater reliability across the 14 codes (unweighted by source size) was above 90%, with approximately 5% disagreement and a kappa of .65 [14,15]. In cases of disagreement, designations from each researcher were included. After the initial round of coding, second cycle coding methods included further coding within specific categories, as well as axial coding, in which data was assembled into categories and dimensions of categories identified [16,17], in accordance with the primary research questions.

### Eligibility

A total of 354 males were screened, of which 128 met eligibility criteria and 48 completed the interviews. Primary reasons for ineligibility included no travel outside of Kazakhstan in the past year (n=144, 40.68%), no permanent residence outside of Kazakhstan or Almaty (n=107, 30.23%), and no vaginal or anal sex with a female partner in the previous 90 days (n=73, 20.62%). Few participants were ineligible due to their age (n=2, 0.56%) or not being currently employed at a stall (n=1, 0.28%). Participants could be ineligible for multiple reasons.

## Results

### Socio-demographic characteristics

Participants’ average age was 28, and the sample included 12 Kazakh (internal migrants), 13 Kyrgyz, 12 Tajik, and 11 Uzbek participants (Table 1). Participants also represented a range of ethnicities in addition to these nationalities: five men from Kazakhstan were ethnically Uighur, four men from Kazakhstan and one from Kyrgyzstan were Dungan, and five men from Uzbekistan were Karakalpak. About 60% of the participants were married (n=29, 60.42%), and most of these men lived with their wives (n=22). Three men reported visiting their wives weekly, and four men visited less frequently. Thirty-three men (68.75%) had either completed secondary school (n=11) or higher education (n=22). The majority of participants were Muslim (n=45, 93.75%). The average length of time the men had worked in Baraholka Market was 4.4 years (SD=3.6 years).

Living arrangements, employment, migration, mobility, and social structural context

In this section, we describe living conditions, employment, mobility, and police harassment, which have been found in other studies to influence HIV risk among migrant workers [6,8].

#### Living arrangements and conditions:

Nearly all study participants lived with their family, relatives, or friends in an apartment or house, while three men lived alone. Most men said they were satisfied with their living conditions, although some phrased their perspective as participant #9 (an internal migrant who lived with his wife in a temporary construction), “as people would say, we are surviving,” or, “it is normal.” Several respondents noted that they lacked basic amenities, such as an indoor toilet or gas for heat or cooking in the home. Participant #5, who lived with his wife in a one-room apartment, said, “…it is difficult to find a good, normal apartment. There is only one room and that’s it, no amenities… just a room and that’s all.”

#### Employment context:

Employment at the marketplace was generally secured through the assistance of a friend, acquaintance, or relative. Most workers had legal registration, which can be obtained through the immigration office or from the migration police. Although most respondents reported being content with their working conditions, some talked about challenges. For example, participant #39, who spent up to 70% of his salary on rent, described, “I am hungry if I miss my work even for one day. It is a struggle for survival here.” However, Almaty was generally described as a place of economic opportunity in comparison with their home communities, which were predominantly located in rural areas.

Most men indicated that they were sending a portion of the money they earned to their families or relatives. Men talked about sending remittances back home, for example, “I send money to my parents when they ask me for it. Once, they needed money urgently. So they asked me if I could help them. I mostly earn a living for my wife and myself” (participant #9). Several participants noted that the ability to earn money for their families was a key motivation for seeking economic opportunity in Kazakhstan. One man (#27) described, “I do not have a father. My mother lives in Tajikistan. I support my mother and my relatives by working here and sending them money. We must live.”

Accounts suggested that some men were willing to perform work they considered beneath them. For example, a 35 year-old man who had migrated to Kazakhstan as a child to escape the civil war in Tajikistan (participant #22) said, “We did not have higher education. That is why we had to look for a job in the market… That is why I have to do such work… to support my family.” Participants appeared to make social and personal sacrifices in the process of migration, in order to achieve both immediate economic benefits and to ensure their ability to send remittances home.

#### Mobility:

Mobility was frequent due to Kazakhstan’s proximity to other countries, especially Kyrgyzstan. Although a few participants traveled as often as weekly or monthly, it was most common to report traveling approximately once a year, and trips generally lasted for less than a week. Seven men had previously worked in Russia. Men from Kyrgyzstan most commonly reported traveling, likely due to close proximity between Kazakhstan and Kyrgyzstan, followed by those from Uzbekistan and Tajikistan, and Kazakh internal migrants. Travel destinations included Kyrgyzstan, Tajikistan, China, Russia, Turkey, and other countries. The majority of men who had travelled to China were citizens of Kazakhstan, but ethnically Uighur. Visiting family and friends was the primary reason participants travelled. Most participants travelled to purchase goods and some travelled to sell goods.

#### Police harassment:

**H**arassment by migration police, including raids, detention, and demanding bribes, was reported by nearly one third of participants. Participant #1 described, “If the patrol arrests a person, they do not do anything to that person except to demand money. They only frighten to get the money… If I pay them, they will release me. If not, I will have to stay there for some time.” Some participants experienced difficulties when crossing the border into Kazakhstan. Border police, like migration police, also may ask for bribes or harass migrants, as participant #19 described, “They do not put a stamp if we do not pay a bribe. They find different reasons to create a problem.” Some men expressed fear, saying they avoided going to Almaty or were hesitant to work in Kazakhstan due to migration police.

### Sexual risk behaviors

In this section we discuss sexual risk behaviors including having multiple sexual partners, casual and commercial sexual relationships, sexually transmitted infections, and condom use.

#### Sexual partners:

Slightly more than half of the married participants reported having sex with a partner in addition to their wife. Participants provided a number of justifications for sex with partners outside of their marriage, including: 1) sexual desires when wives are unable to have sex, 2) mobility and separation, 3) need for variety in partners and types of sex, and 4) lack of satisfaction in marriage. Male sexual desire and needs were used as rationalization for both infidelity and intercourse with sex workers.

#### Sexual desires:

Three men talked about sexual needs for an outside partner if their wife could not have sex due to being sick, pregnant, or recently giving birth. For example, participant #13 said “…when a wife is pregnant, a couple cannot have intimacy for 3–4 months. A man cannot wait for a long time and he goes to other women for sex.” Additionally, participant #28 described that “If my wife is sick, I will wait for two weeks. If I cannot control myself, I will find a woman.”

#### Mobility:

Travelling for extended periods of time justified having an outside relationship for some participants. Participant #13 said, “What else can he do if he is on a business trip for a month? He may meet a woman who will care about him (do the laundry for him) and have a sexual relationship with him… She would play the role of a second inconspicuous wife.” Others talked about the ability of some men to abstain despite these desires, for example, “Some married men who have to live far from their families for a while go to women for sex. They have such desire. Some men control themselves in spite of this desire” (participant #27). A few men described meeting casual partners or commercial sex partners while traveling, including visiting Chinese bathhouses. One Kyrgyz migrant (participant #13) said a casual partner can help a man address his basic needs when traveling for a prolonged periods, while participant #18 said the length of time traveling is a consideration, “If I go for one day, it doesn’t happen… If I go for [a] longer time, of course [it] happen[s].”

#### Need for variety:

A few men used metaphors based on food or drink to describe a need for variety as motivating infidelity, such as, “I am sometimes bored of routine and seek something new. For example, when you eat the same food every day, you get tired of it and want to try some other food,” (participant #37) and “As they say you won’t drink tea every day, would you? It is necessary sometimes to have coffee as well (laughter).”

Most participants described only engaging in vaginal sex with their wives or other partners, and a few spoke of a desire to try other forms of sex as a motivator for seeking outside relationships. The degree to which sexual practices were accepted was attributed to religion and culture, with men citing religious proscriptions against anal sex or any sexual practice besides vaginal intercourse. Participants pointed to differences between sexual behaviors with their wives versus with casual or commercial partners. For example, participant #5 described, “With the wife you do it as with the wife. You pay the girl – you can poke where you want.” Another participant (#34) attributed infidelity to interest in experimentation, stating, “They [married men] may want to try other kinds of sex with other women.”

#### Satisfaction in marriage:

Men emphasized the importance of happiness and sexual satisfaction in marriage, and some suggested infidelity may be justified, at least in part, when conflicts arise or expectations are unmet. For example, participant 15 described, “If a man does not love his wife, scolds her and fights with her, he is likely to have a relationship with another woman.” Alternately, participant #12 said, “If a man has a wife and loves her, he will not meet with other women,” attributing fidelity to love. One participant (#36), whose only partner had been his wife, said “I believe that a good, beautiful wife always tries to care about her husband and please him in terms of sex and family life. I do not understand those men who are not faithful to their wives if there are not any objective reasons.” This quote suggests the participant may tolerate infidelity if there were reasons to seek an outside partner. Going further, participant #15 said, “Some women push their husbands to relationships with other women.” Additionally, one participant (#43) described outside relationships as beneficial to the marriage, as, “In my opinion, when a man has sexual relationships with other women, he begins to understand and value his wife and family more.” However, he cautioned, “…family life should not be endangered by continuous infidelity.” Most participants promoted fidelity from both partners as the ideal, for example, stating, “If his behavior is immoral, his wife may also do wrong things. On the contrary, if the partners love each other, they can avoid such problems” (participant #11). Infidelity on the part of wives was less tolerable than for husbands, as respondent #11 described, “A woman is more vulnerable to being criticized than a man.”

#### Casual and non-marital partners:

Participants differentiated between commercial sex and sex with girlfriends or casual partners, where various levels of emotional connection were common among the latter but not with commercial sex workers. Men described meeting casual partners through friends at social events or at the market. A few men commented that it is acceptable to engage in relationships with certain women, such as divorced women, but not with never-married women. None of the respondents reported having sex with men and many expressed views that sex with men would be unacceptable.

Some men said they do not discuss relationships with friends, and a few said their friends encouraged infidelity, as, “my friends supported my wish to meet that girl and continue our communication so that [I could] have sex with her. Nobody dissuaded me” (participant #3, who had a girlfriend but was unmarried). Others said that friends played a role in encouraging fidelity as important for family life. Participant #13 said he would warn someone against cheating on his wife, “If he wants to have sex with other women, he can do it but the consequences can be horrible.”

#### Commercial sex:

Nearly half of respondents reported having sex with a commercial partner at least once, and many of these had sex with a commercial partner more recently or regularly. Saunas were primary venues where men met commercial partners, and many participants were aware of particular streets in Almaty where sex workers congregated. Commercial sex was perceived as cheaper outside of Almaty, and men reported having sex with commercial partners while travelling or visiting their home communities. Participant #11described commercial sex as something interesting for men who have recently migrated, as “They can go to such women to learn what it is like for the first time.” Attitudes towards commercial partners were largely negative, suggesting engagement in these relationships challenges conceptions of appropriate masculinity. For example, “I went to a prostitute once. I paid money for sex but I did not feel well after having sex with her. I was disgusted… I washed my clothes and myself. I have not gone to prostitutes since then (Participant #1).”

In contexts of commercial sex, men place responsibility on women to protect themselves and their clients. Participants commented on the responsibility of women (including sex workers or women with many partners) for the transmission of sexually transmitted infections, as “women have infections that adversely affect men’s health,” (participant #41) and “I protect myself from diseases that women can have” (participant #39).

Meeting with sex workers was often a social activity involving male friendships, as men described visiting commercial sex establishments or streets where sex workers are known to be with other men. Participant #31 described a group encounter with a sex worker, where a driver brought one woman who was shared between 25–30 men over the course of 3–4 hours. He said he did not use a condom. This situation highlights extreme levels of STI and HIV risk.

#### Sexually Transmitted Infections (STIs):

While a number of men said they knew someone who had an STI, only participant #5 said he had an STI after an unprotected encounter with a commercial sex worker and received treatment. It is possible that shame associated with having an STI may have made some men feel uncomfortable disclosing this information. Those who engaged in extra-marital sexual relationships expressed discomfort about such behaviors and expressed an obligation to protect their wives, suggesting conceptions of masculine familial responsibility may conflict with masculine sexual identity. None of the men reported intentions to protect sex workers or casual partners from HIV or STIs. A few men described the impact STIs could have on their families, and their efforts to take protective measures. Participant #34 said someone he knew “slept with his wife after having sex with prostitutes and his wife got sick too. Therefore, I use condoms to avoid sexually transmitted infections” and another participant (#33) said, with an eye towards future relationships, “I would not want to infect my future wife and children with any disease.” By displaying concern for their wives and families, participants enact a masculine role as a protector of their family.

#### Condom use:

Men identified types of partners, trust, sexual pleasure, health protection, and alcohol use as factors influencing condom use.

#### Type of partners:

While most men in this study said they used condoms with casual partners, many described exceptions or using condoms only during the initial sexual encounter. Less than half had used condoms with their spouse, and condoms were generally only used to prevent pregnancy. A few men were skeptical about the ability of condoms to prevent disease, as “I think that infections can penetrate through condoms” (participant #35).

#### Trust:

Trust was a large factor in men’s decisions regarding condom use. Those who chose not to use condoms talked about “being confident about my partners,” or mutual trust, where “we trusted each other.” Familiarity was also a component of the decision, “if I had sex with a stranger I had to use them [condoms].” Trust may be built upon perceptions of cleanliness and a partner’s characteristics including her job or lifestyle. Men talked about not trusting women because they were unmarried, drink alcohol, or agree to have sex. For example, “I did not trust them because they were single, rented apartments, and lived separately from their parents” (participant #19). Men also described the role of reported or apparent health when deciding whether or not to use a condom, saying they would not use a condom if the woman looked healthy or if she says she is healthy [18].

#### Sexual pleasure:

A third of respondents said condoms reduced pleasure. Despite some knowledge regarding the protective benefits of condoms and accessibility of condoms, concerns about reduction in pleasure and confidence that partners are trustworthy puts men at risk of acquiring STIs [18,19]. As one participant (#31) related, “I wanted to be with the woman very much. When a man desires a woman, he does not pay attention to safety. He does not think about being infected with diseases.”

#### Health protection:

Some men talked about protecting their wives, for example, “I used contraceptive sheaths when I had sex with other women. I have a family and did not want my wife to get sick” (participant #22). Men also talked about responsibility for their own health, for example, “A person should take care of himself/herself… A person must always have condoms when he/she intends to have sex with someone” (participant #1). A few men talked about receiving encouragement from friends to use condoms or to be aware of their health.

#### Alcohol use:

Respondents reported that they were less likely to use condoms when consuming alcohol, as “I forgot about using contraceptive sheaths because I was drunk” (participant #20) or “I remember one case when I was very drunk. I did not remember if I used contraceptive sheaths or not. But the girl was not a prostitute” (participant #3).

## HIV/AIDS knowledge

Participants demonstrated low levels of knowledge about HIV/AIDS and believed HIV was not affecting their community. Participant #3 said, “I do not think it [HIV] is a problem. I do not know anyone who suffers from this disease. I have heard about it only on television.” No participants knew someone infected with HIV and some participants had never heard of HIV/AIDS. The primary source of information about HIV was television programming, while a number also mentioned receiving information from school, newspapers, and clinics. One participant (#3) described seeing information on posters in the hospital but not being able to read Russian, suggesting the need for language-specific materials to reach some migrants. The majority of participants were aware that HIV is transmitted sexually, but misunderstanding of modes of HIV acquisition was common, with transmission attributed to kissing, shared dishes, saliva, touch, a lack of hygiene, washing together, or damp air. Some participants said they would rely on their own or their partner’s judgment to determine whether a person had HIV. Additionally, a few participants felt they would “know” if they were sick. Only two respondents specified that HIV may be asymptomatic and testing is necessary to diagnose the disease.

## HIV testing

Less than half of participants reported ever receiving an HIV test. Of these, most were tested as part of a general medical exam. Others had been tested as part of army service, in relation to a spouse’s pregnancy, or through an NGO. Some participants were unsure whether they had received the test or what the results were. Few men sought out testing due to a concern for their sexual health.

Of those who had not been tested, most felt they were healthy and not at risk. For example, a 23 year-old migrant with several casual partners (#19) said, “I have not had any problems, and, therefore, I have not taken it. I meet with normal girls.” Participant #16, whose only sexual partner was his wife, said, “If I were suspicious about being sick, I would undergo medical examination.” Describing why others do not get tested, participant #3 reported, “They think everything is fine. They do not know where they can take these tests. They do not think about the danger. They do not know about the danger.” Men also attributed testing avoidance to shame.

Some participants said HIV testing was a required component of registration and citizenship documentation in Kazakhstan as well as in neighboring countries. While testing may be required for those migrating legally, others may avoid testing due to migration related concerns. A married migrant with several casual partners (participant #17) said, “I did not want to take an HIV test at the beginning.” When asked why, he laughed, saying, “I do not know. I live illegally here,” suggesting that testing may jeopardize one’s hidden migration status.

## Access to health care services

Cost and migration status were key barriers to accessing adequate health care services. A number of respondents said they sought health care in their hometowns, while many sought care in private clinics in Kazakhstan and few received care from public clinics, which primarily serve citizens or registered migrants. Some men had used emergency services or an ambulance, but one migrant described being refused services and sent home because he was a foreigner and unable to pay. Some men said their wives registered at local maternity clinics when pregnant or had children who were registered at children’s clinics, as all children and pregnant women have access to medical care in Kazakhstan regardless of legal status. A few men discussed seeking advice from pharmacists or described consulting folk medicine.

In addition to cost barriers to health services, respondents said immigration status was a barrier to care. In response to access barriers, participants described the role of bribes. A lack of power and resources appears to limit men’s ability to ensure access to necessary health care services for themselves and their families.

Social networks play a critical role in the sharing of health information, as participants said that they receive health information from friends, family, and acquaintances. Men discussed informing their friends about what they learned from doctors, healing themselves by listening to other people’s advice, and passing information from older to younger generations. Participant #30, a 37 year-old said, “… if I am sick, I’ll go to the doctor, then if my friend is sick, I’ll tell him what to do. So in that way we learn from each other.” Participant #33 said he consulted with doctors, but also said, “Being Muslims, we heal ourselves by listening to other people’s advice. We buy medicine that people advise [us] to take.” The role of women was noted, as participant #39 described, “Women mostly know well how to treat diseases and they can give some good pieces of advice.”

## Discussion

The findings from this qualitative study provide a description of the contexts that drive sexual risk behaviors (commercial sex, casual sex, condom use), HIV knowledge, HIV testing, and access to health care among male migrant vendors from Central Asia who work in the largest market place in Kazakhstan.

Masculinity is heavily influenced by factors such as age, social class, ethnicity, and sexual orientation [20]. The men in this study may be in a subordinated or vulnerable position due to their migrant status. Many participants reported harsh living conditions and harassment by immigration police, all of which may challenge conceptions of masculine identity. Despite the challenging conditions they experience, most participants found purpose in exhibiting masculine characteristics in other roles such as providing economic support for their families. Connell has labeled this behavior multiple masculinities or “flexible and strategic masculinities [9].” This situation is consistent with hegemonic masculinity theory, underscoring requirements that men fulfill specific duties and obligations, while embracing what is expected from them by supporting immediate and often extended family members [9].

Respondents tended to engage in vaginal sex with their wives but were more likely to report anal or oral sex with other partners. With commercial and casual partners, some men may allow themselves to experiment and practice different types of sex. Commercial sex may be justified by hegemonic masculinity accounts, as a number of participants believed they have sexual needs that must be fulfilled when their spouse is not available; however, seeking other partners is less tolerable for women. This account is also supported by male oriented gender and cultural expectations. Women are required to follow specific cultural expectations such as having sex only with their husband, where men’s outside sexual relationships are more likely to be tolerated.

Condom use was reported with casual and commercial partners, but was less common within marriage. These findings support earlier research findings showing men tend to use condoms with commercial sex partners, and use condoms less often with long-term partners [21–23]. Men used the notion of trust to justify their lack of condom use in marriage and with some outside partners, describing their wives as clean and trusting they would not put their husband at risks.

Participants had poor knowledge of HIV/AIDS and did not believe HIV was affecting their social or sexual networks in the marketplace, although a number of men reported learning about HIV/AIDS through their social networks and other health or media venues. Consistent with prior research, factors identified as barriers to HIV testing included misperceptions about HIV, lack of knowledge on the importance of knowing one’s HIV status, lack of access to testing, and fears of deportation [24–27]. These factors have been found in other studies among vulnerable groups as barriers to HIV testing [6,28–30]. Men’s lack of health help-seeking behaviors has been documented in literature worldwide [31] and has been attributed to attempts at enacting masculine ideals [20]. By rejecting health care, men demonstrate their beliefs in their physical strength and self-reliance. A behavior such as HIV testing may be considered to be at-odds with behaviors that demonstrate masculinity and sexual prowess, such as having multiple partners and condom non-use. Participants’ dismissal of the importance of HIV testing, and the discomfort some men felt by seeking testing, combined with concerns for the health and well-being of their families shows how their behavior is influenced by the need to enact multiple masculinities.

This study has a number of limitations. We recruited participants through a convenience sampling approach, where men self-selected to be included in the study. This may limit the generalizability of qualitative study findings to other Central Asian male migrants. Although research staff highlighted study protocols related to confidentiality and anonymity of reported data, participants may have had difficulty disclosing sensitive information such as sexual behavior and illegal status. Additionally, interviewers were not matched with participants by gender, with interviews conducted primarily by female researchers.

However, researchers stressed the confidentiality of the research throughout, and encouraged participants to be open and honest, pointing to a number of strengths of the study. We believe participants openly presented their challenges and circumstances regarding sexual risk behavior and other sensitive topics including relationships and contexts of migration. Interviews were conducted privately and in multiple languages, and the comprehensive nature of these interviews allowed us to examine a variety of themes related to HIV risk.

## Conclusion

Frequent mobility and sexual mixing between Almaty and rural destinations in Kazakhstan or in other countries may facilitate the spread of HIV and sexually transmitted infections to lower endemic areas. Similarly, sexual mixing while buying goods from foreign locations may result in transporting HIV to sexual partners in Almaty or to partners in the country of origin. The high level of risky sexual behaviors found among this population of migrant workers coupled with the low level of knowledge about HIV/AIDS and lack of access to HIV/STI prevention services call for attention to address potential HIV transmission.

The marketplace provides a unique venue to deliver prevention messages through a number of possible strategies including media campaigns to promote HIV/STI prevention; opening a health clinic or mobile health van in the marketplace that provides HIV/STI prevention, testing, and treatment services; delivering HIV prevention messages through peer-led social network approaches; distributing free condoms at the marketplace; and providing free access to confidential and anonymous HIV/STI testing. These strategies must be delivered with recognition of gender based and culturally congruent approaches and attention to how men variants masculinities in the context of migration and sexual HIV risk. Messages included in HIV prevention strategies must contextualize risk reduction messages by integrating and demonstrating how masculine norms, gender expectations, and the meanings men attach to their sexual risk behaviors are barriers for sexual risk reduction.

## Figures and Tables

**Table 1:** Socio-demographic characteristics of 48 male migrants.

Age in years (mean, SD)	28	7.6
Nationality		
Kazakh (Uighur: 5, Dungan: 4)	12	25.0%
Kyrgyz (Dungan: 1)	13	27.1%
Tajik	12	25.0%
Uzbek (Karakalpak: 5)	11	22.9%
Marital Status		
Married	29	60.4%
Single	16	33.3%
Divorced	3	6.3%
Household status among married men (n=29)		
Lives with wife	24	82.8%
Visits wife weekly	2	6.9%
Visits less frequently	3	10.3%
Education		
Less than secondary school	7	14.6%
Secondary school	11	22.9%
University/vocational school	22	45.8%
Not specified	8	16.7%
Religion		
Islam	45	93.8%
Not religious	2	4.1%
Not discussed	1	2.1%
Time working in Baraholka market (mean, SD)	4.4	3.6%
Living conditions		
Lives in an apartment	24	50.0%
Lives in a house	8	16.7%
Lives in a single room in a house or apartment	12	25.0%
Lives in a one room extension or temporary construction	4	8.3%
